# The genome sequence of a hoverfly, *Merodon equestris* (Fabricius, 1794)

**DOI:** 10.12688/wellcomeopenres.20654.1

**Published:** 2024-02-19

**Authors:** Olga Sivell, Liam M. Crowley, Natural History Museum Genome Acquisition Lab, University of Oxford and Wytham Woods Genome Acquisition Lab, Darwin Tree of Life Barcoding collective, Wellcome Sanger Institute Tree of Life Management, Samples and Laboratory team, Wellcome Sanger Institute Scientific Operations: Sequencing Operations, Wellcome Sanger Institute Tree of Life Core Informatics team, Tree of Life Core Informatics collective, Darwin Tree of Life Consortium

**Affiliations:** 1 Natural History Museum, London, England, UK; 2 University of Oxford, Oxford, England, UK

**Keywords:** Merodon equestris, hoverfly, genome sequence, chromosomal, Diptera

## Abstract

We present a genome assembly from an individual female *Merodon equestris* (hoverfly; Arthropoda; Insecta; Diptera; Syrphidae). The genome sequence is 873.0 megabases in span. Most of the assembly is scaffolded into 6 chromosomal pseudomolecules. The mitochondrial genome has also been assembled and is 15.95 kilobases in length.

## Species taxonomy

Eukaryota; Metazoa; Eumetazoa; Bilateria; Protostomia; Ecdysozoa; Panarthropoda; Arthropoda; Mandibulata; Pancrustacea; Hexapoda; Insecta; Dicondylia; Pterygota; Neoptera; Endopterygota; Diptera; Brachycera; Muscomorpha; Eremoneura; Cyclorrhapha; Aschiza; Syrphoidea; Syrphidae; Eristalinae; Merodontini; *Merodon*; *Merodon equestris* (Fabricius, 1794) (NCBI:txid511117).

## Background

*Merodon equestris* (Fabricius, 1794) is a large, hairy, bumblebee-mimicking hoverfly with a small head and somewhat elongate abdomen as compared to *Bombus* species. The species is commonly called the Large Bulb Fly or Large Narcissus Fly, as the larvae are pests of flower bulbs. It is the only species from the genus *Merodon* occurring in Britain ( Ball & Morris, 2015; Chandler, 2023). It can be easily identified by the clear wing, a loop in vein R4+5, black hind legs with swollen femora with a large triangular ventral projection ( Ball & Morris, 2015; Stubbs & Falk, 2002; van Veen, 2014). There are 34 colour types, e.g. thorax black or tawny haired, or tawny anteriorly and black posteriorly and abdomen with a grey, yellow, orange, whitish, buff, or red tail; or abdomen tawny-haired with a transverse black band on tergite 3 ( Conn, 1972; Stubbs & Falk, 2002).

*Meredon equestris* is a Palaearctic species occurring from Fennoscandia to the Mediterranean, European parts of Russia, North Africa, Japan, North America and it has been introduced to New Zealand ( Speight, 2010) and recently reported from South Korea ( Han *et al.*, 2018). It has been recorded from Britain since 1869. It is believed to have been introduced from The Netherlands with narcissus bulbs ( Hodson, 1932). It is now widely distributed in Britain and Ireland, and can be found in gardens, urban sites and countryside, with adults on the wing from April to September ( Ball *et al.*, 2011; Ball & Morris, 2015; Stubbs & Falk, 2002).

The larvae feed on a number of bulb-producing plant species such as *Narcissus*, *Hyacinth*, *Tulipa* (rarely), *Amaryllis*, *Habranthus*, *Vallota*, *Galtonia*, *Scilla*, *Leucojum*, *Eurycles*, the wild snowdrop *Galanthus* and wild bluebell *Hyacinthoides non-scripta* ( Fryer, 1914; Hodson, 1932). The eggs are laid on a leaf close to the ground or on a flower bulb ( Hodson, 1932; Stubbs & Falk, 2002). They hatch after approximately 10 to 15 days. The newly hatched larva is distinctively different in appearance from later stages. It usually enters the bulb through the thinner base and begins feeding. Typically, one larva is found in a bulb, and it can migrate to another bulb if the food is depleted and the bulbs are grown closely together. The larval stage lasts approximately 300 days and larva vacates the bulb in early spring, digs through the soil and pupates at the surface level or just below it. The pupal stage lasts approximately 35 to 40 days. The observed adult lifespan was between 5 and 24 days for females and 6 to 18 days for males ( Hodson, 1932). The males are aggressive in copulation and will attempt to engage with anything resembling a female, including bumblebees and other males. If successful the flies will land (if flying) and copulate on the ground ( Conn, 1971). Adults fly low, often rest on the ground, and can be encountered feeding on umbellifers ( Speight, 2017).

There is one generation per year and the species overwinters as a larva inside the plant bulb ( Conn, 1971; Hodson, 1932). The egg, larva and pupa were described by Hodson (1932), the larva was also described by Heiss ( Heiss, 1938) and was later illustrated by Rotheray (1986). The genetics of *Merodon equestris* colour polymorphism have been studied by Conn ( 1971; 1972; 1976).

The high-quality genome sequence described here is the first one reported for *Merodon equestris.* It was sequenced based on one specimen from the Eden Project, England. It will aid research on biology, phylogeny and ecology of the species. This genome has been generated as part of the Darwin Tree of Life Project, a collaborative effort to sequence all named eukaryotic species in the Atlantic Archipelago of Britain and Ireland.

## Genome sequence report

**Figure 1.**
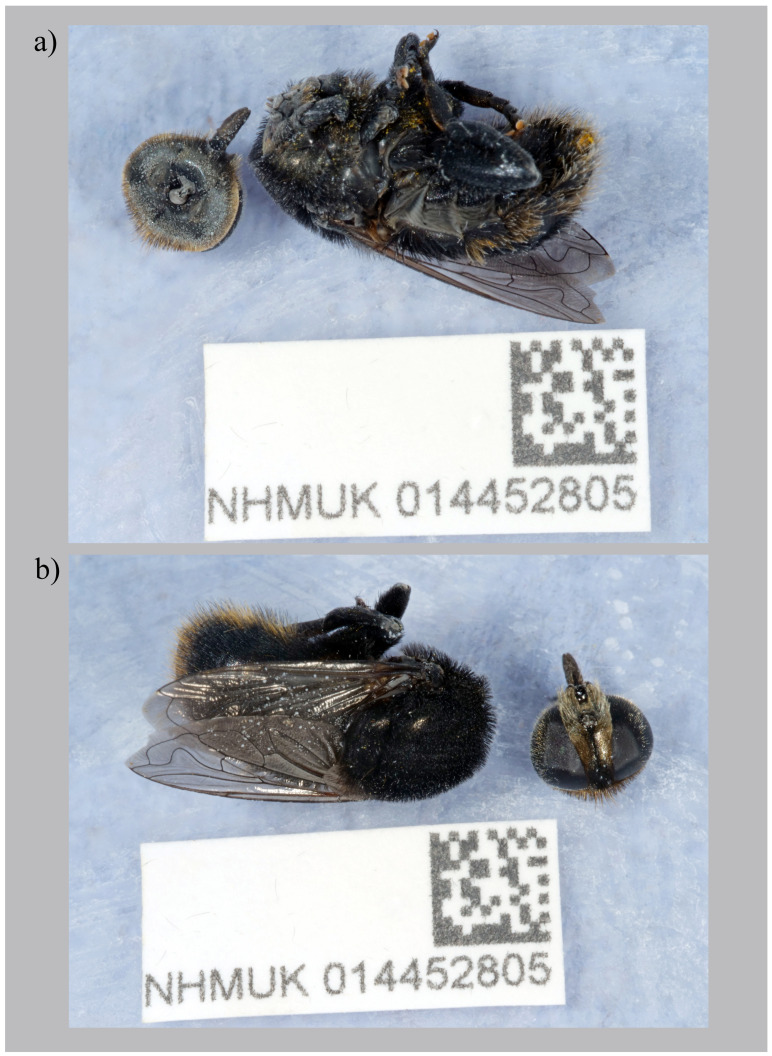
*Merodon equestris* (Fabricius, 1794), specimen used for genome sequencing (NHMUK014452805) **a**) body in latero-ventral view, head in ventral view; **b**) body in dorsal view, head in anterior view.

The genome was sequenced from one *Merodon equestris* ( Figure 1) collected from The Eden Project in Cornwall (50.36, –4.74). A total of 33-fold coverage in Pacific Biosciences single-molecule HiFi long reads was generated. Primary assembly contigs were scaffolded with chromosome conformation Hi-C data. Manual assembly curation corrected 44 missing joins or mis-joins and removed 5 haplotypic duplications, reducing the scaffold number by 6.21%, and increasing the scaffold N50 by 3.69%.

**Figure 2.**
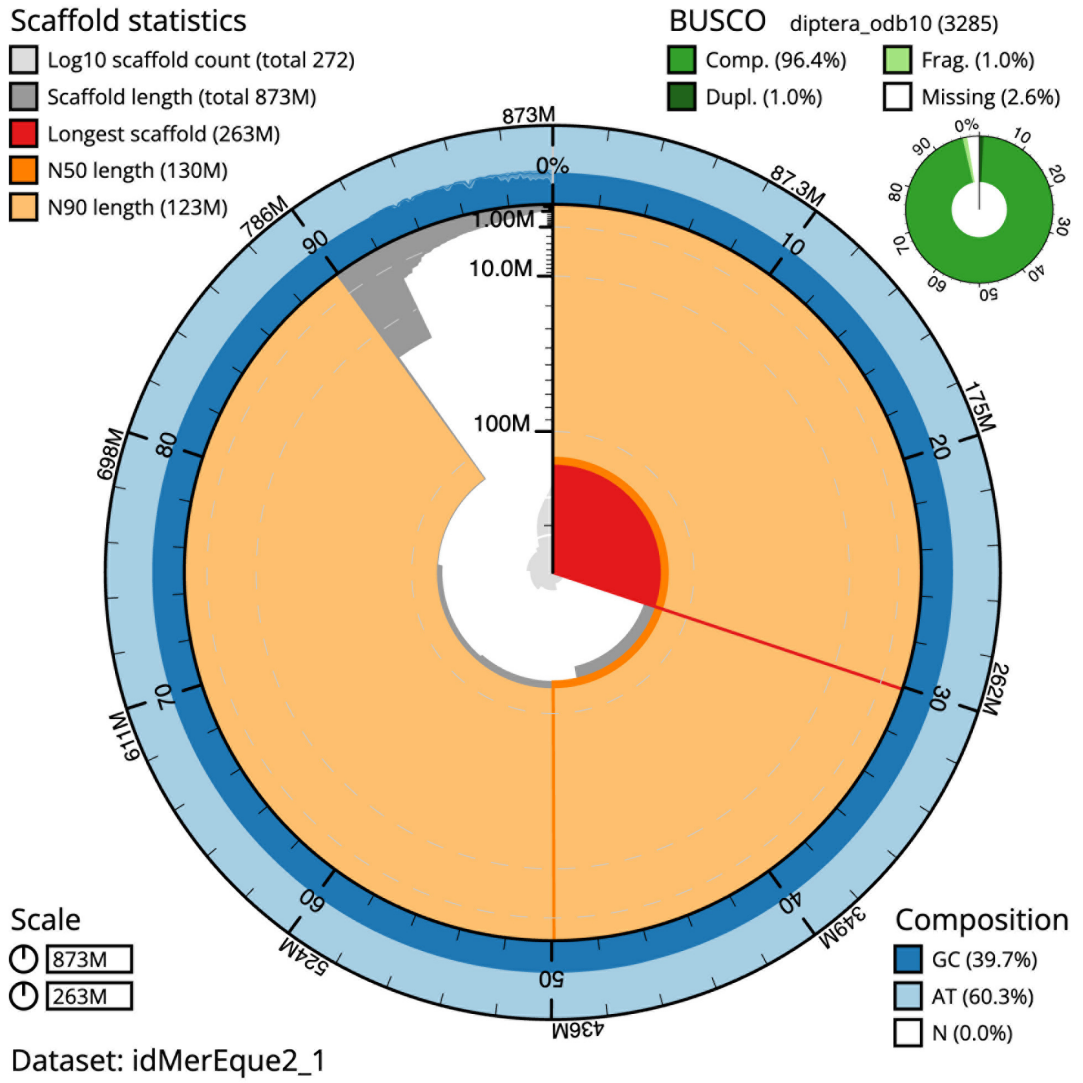
Genome assembly of *Merodon equestris*, idMerEque2.1: metrics. The BlobToolKit Snailplot shows N50 metrics and BUSCO gene completeness. The main plot is divided into 1,000 size-ordered bins around the circumference with each bin representing 0.1% of the 872,988,745 bp assembly. The distribution of scaffold lengths is shown in dark grey with the plot radius scaled to the longest scaffold present in the assembly (262,796,939 bp, shown in red). Orange and pale-orange arcs show the N50 and N90 scaffold lengths (129,753,689 and 123,427,599 bp), respectively. The pale grey spiral shows the cumulative scaffold count on a log scale with white scale lines showing successive orders of magnitude. The blue and pale-blue area around the outside of the plot shows the distribution of GC, AT and N percentages in the same bins as the inner plot. A summary of complete, fragmented, duplicated and missing BUSCO genes in the diptera_odb10 set is shown in the top right. An interactive version of this figure is available at https://blobtoolkit.genomehubs.org/view/idMerEque2_1/dataset/idMerEque2_1/snail.

**Figure 3.**
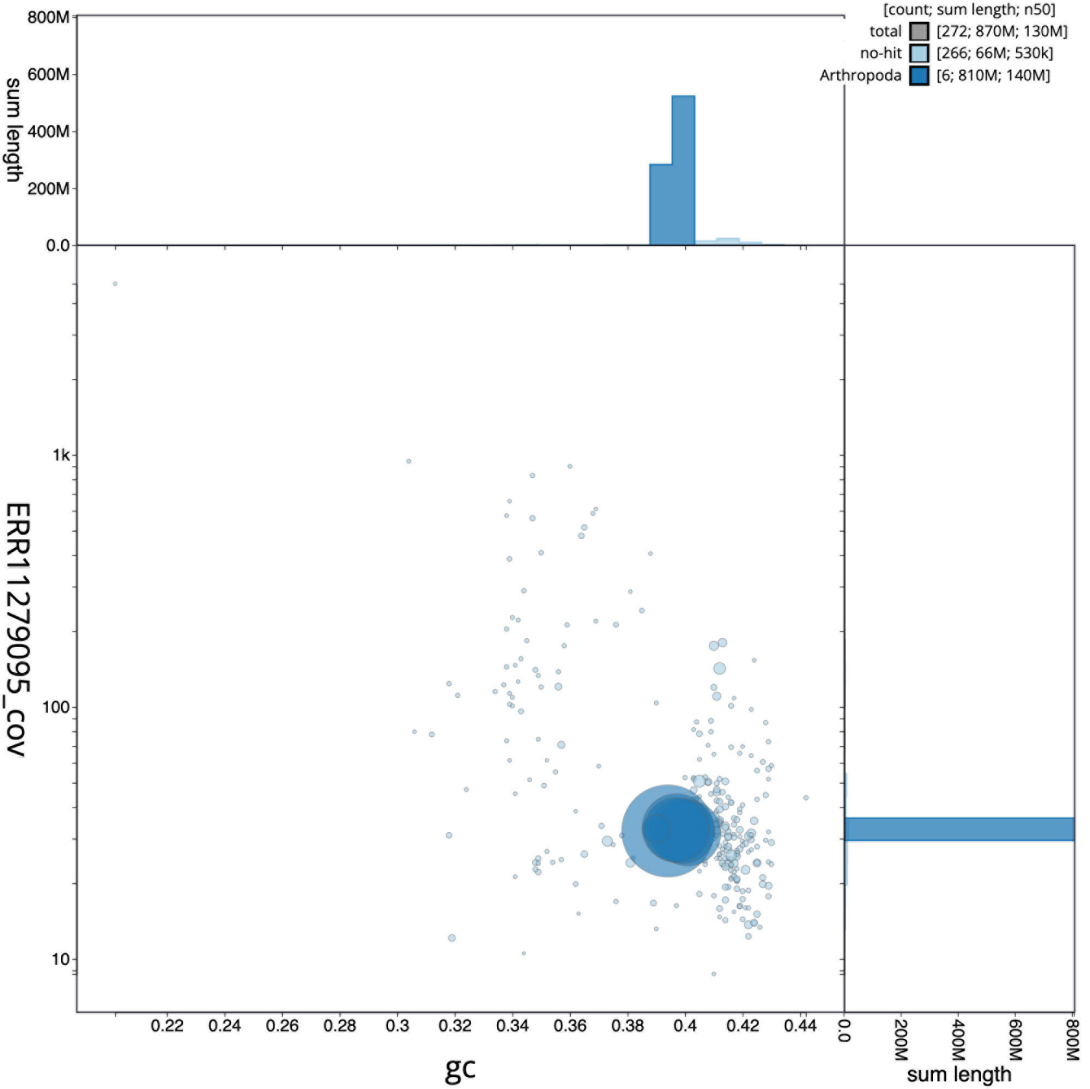
Genome assembly of *Merodon equestris*, idMerEque2.1: BlobToolKit GC-coverage plot. Scaffolds are coloured by phylum. Circles are sized in proportion to scaffold length. Histograms show the distribution of scaffold length sum along each axis. An interactive version of this figure is available at https://blobtoolkit.genomehubs.org/view/idMerEque2_1/dataset/idMerEque2_1/blob.

**Figure 4.**
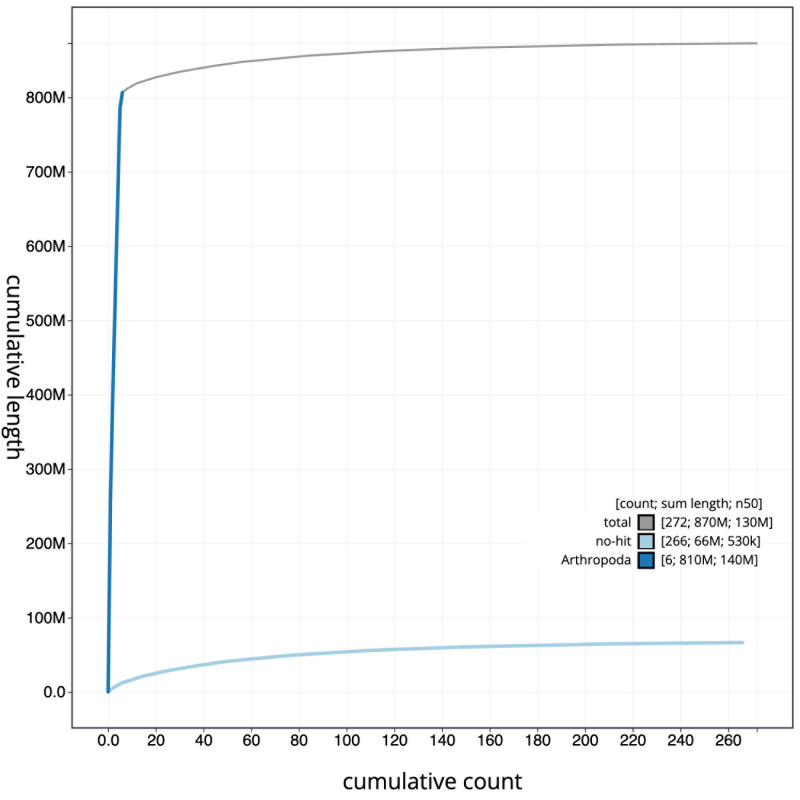
Genome assembly of *Merodon equestris*, idMerEque2.1: BlobToolKit cumulative sequence plot. The grey line shows cumulative length for all scaffolds. Coloured lines show cumulative lengths of scaffolds assigned to each phylum using the buscogenes taxrule. An interactive version of this figure is available at https://blobtoolkit.genomehubs.org/view/idMerEque2_1/dataset/idMerEque2_1/cumulative.

**Figure 5.**
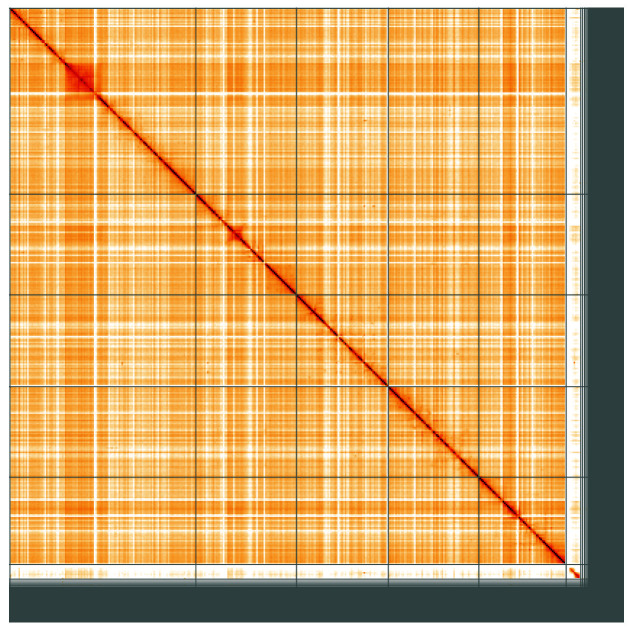
Genome assembly of *Merodon equestris*, idMerEque2.1: Hi-C contact map of the idMerEque2.1 assembly, visualised using HiGlass. Chromosomes are shown in order of size from left to right and top to bottom. An interactive version of this figure may be viewed at https://genome-note-higlass.tol.sanger.ac.uk/l/?d=GJMrxZniSRaNqvLTHHNs2w.

The final assembly has a total length of 873.0 Mb in 271 sequence scaffolds with a scaffold N50 of 129.8 Mb ( Table 1). The snailplot in Figure 2 provides a summary of the assembly statistics, while the distribution of assembly scaffolds on GC proportion and coverage is shown in Figure 3. The cumulative assembly plot in Figure 4 shows curves for subsets of scaffolds assigned to different phyla. Most (92.4%) of the assembly sequence was assigned to 6 chromosomal-level scaffolds. Chromosome-scale scaffolds confirmed by the Hi-C data are named in order of size ( Figure 5; Table 2). While not fully phased, the assembly deposited is of one haplotype. Contigs corresponding to the second haplotype have also been deposited. The mitochondrial genome was also assembled and can be found as a contig within the multifasta file of the genome submission.

**Table 1.** Genome data for *Merodon equestris*, idMerEque2.1.

Project accession data		
Assembly identifier	idMerEque2.1	
Species	*Merodon equestris*	
Specimen	idMerEque2	
NCBI taxonomy ID	511117	
BioProject	PRJEB61688	
BioSample ID	SAMEA112221777	
Isolate information	idMerEque2, female: thorax (DNA sequencing) idMerEque1: head and thorax (Hi-C sequencing) idMerEque3: abdomen (RNA sequencing)	
Assembly metrics *		*Benchmark*		
Consensus quality (QV)	65.8	*≥ 50*
*k*-mer completeness	100.0%	*≥ 95%*
BUSCO **	C:96.4%[S:95.4%,D:1.0%], F:1.0%,M:2.6%,n:3,285	*C ≥ 95%*
Percentage of assembly mapped to chromosomes	92.4%	*≥ 95%*
Sex chromosomes	Not identified	*localised homologous pairs*
Organelles	Mitochondrial genome: 15.95 kb	*complete single alleles*
Raw data accessions		
PacificBiosciences SEQUEL II	ERR11279095	
Hi-C Illumina	ERR11439617	
PolyA RNA-Seq Illumina	ERR11837489	
Genome assembly		
Assembly accession	GCA_958301585.1	
*Accession of alternate haplotype*	GCA_958450385.1	
Span (Mb)	873.0	
Number of contigs	463	
Contig N50 length (Mb)	5.6	
Number of scaffolds	271	
Scaffold N50 length (Mb)	129.8	
Longest scaffold (Mb)	262.8	

* Assembly metric benchmarks are adapted from column VGP-2020 of “Table 1: Proposed standards and metrics for defining genome assembly quality” from ( Rhie *et al.*, 2021). ** BUSCO scores based on the diptera_odb10 BUSCO set using version 5.3.2. C = complete [S = single copy, D = duplicated], F = fragmented, M = missing, n = number of orthologues in comparison. A full set of BUSCO scores is available at https://blobtoolkit.genomehubs.org/view/idMerEque2_1/dataset/idMerEque2_1/busco.

**Table 2.** Chromosomal pseudomolecules in the genome assembly of *Merodon equestris*, idMerEque2.

INSDC accession	Chromosome	Length (Mb)	GC%
OY284413.1	1	262.8	39.5
OY284414.1	2	142.22	39.5
OY284415.1	3	129.75	40.0
OY284416.1	4	127.86	40.0
OY284417.1	5	123.43	39.5
OY284418.1	6	20.61	39.0
OY284419.1	MT	0.02	20.0

The estimated Quality Value (QV) of the final assembly is 65.8 with *k*-mer completeness of 100.0%, and the assembly has a BUSCO v5.3.2 completeness of 96.4% (single = 95.4%, duplicated = 1.0%), using the diptera_odb10 reference set ( *n* = 3,285).

Metadata for specimens, barcode results, spectra estimates, sequencing runs, contaminants and pre-curation assembly statistics are given at https://links.tol.sanger.ac.uk/species/511117.

## Methods

### Sample acquisition and nucleic acid extraction

A specimen *Merodon equestris* specimen used for DNA sequencing (specimen ID NHMUK014452805, ToLID idMerEque2) and a second specimen used for RNA (NHMUK014452803, idMerEque3) were collected at the Eden Project (latitude 50.36, longitude –4.74) on 2021-06-28 using an aerial net. These specimens were collected and identified by Olga Sivell (Natural History Museum, London) and preserved by dry freezing at –80°C. The specimen used for Hi-C sequencing (specimen ID Ox000426, ToLID idMerEque1) was netted in Wytham Woods, Oxfordshire (biological vice-county Berkshire), UK (latitude 51.77, longitude –1.34) on 2020-05-22. The specimen was collected and identified by Liam Crowley (University of Oxford) and preserved on dry ice.

The workflow for high molecular weight (HMW) DNA extraction at the Wellcome Sanger Institute (WSI) includes a sequence of core procedures: sample preparation; sample homogenisation, DNA extraction, fragmentation, and clean-up. In sample preparation, the idMerEque2 sample was weighed and dissected on dry ice ( Jay *et al.*, 2023). Tissue from the thorax was homogenised using a PowerMasher II tissue disruptor ( Denton *et al.*, 2023a). HMW DNA was extracted using the Automated MagAttract v2 protocol ( Oatley *et al.*, 2023a). The DNA was sheared into an average fragment size of 12–20 kb in a Megaruptor 3 system with speed setting 31 ( Bates *et al.*, 2023). Sheared DNA was purified by solid-phase reversible immobilisation ( Oatley *et al.*, 2023b): in brief, the method employs a 1.8X ratio of AMPure PB beads to sample to eliminate shorter fragments and concentrate the DNA. The concentration of the sheared and purified DNA was assessed using a Nanodrop spectrophotometer and Qubit Fluorometer and Qubit dsDNA High Sensitivity Assay kit. Fragment size distribution was evaluated by running the sample on the FemtoPulse system.

RNA was extracted from abdomen tissue of idMerEque3 in the Tree of Life Laboratory at the WSI using the RNA Extraction: Automated MagMax™ *mir*Vana protocol ( do Amaral *et al.*, 2023). The RNA concentration was assessed using a Nanodrop spectrophotometer and a Qubit Fluorometer using the Qubit RNA Broad-Range Assay kit. Analysis of the integrity of the RNA was done using the Agilent RNA 6000 Pico Kit and Eukaryotic Total RNA assay.

Protocols developed by the WSI Tree of Life core laboratory are publicly available on protocols.io ( Denton *et al.*, 2023b).

### Sequencing

Pacific Biosciences HiFi circular consensus DNA sequencing libraries were constructed according to the manufacturers’ instructions. Poly(A) RNA-Seq libraries were constructed using the NEB Ultra II RNA Library Prep kit. DNA and RNA sequencing was performed by the Scientific Operations core at the WSI on Pacific Biosciences SEQUEL II (HiFi) and Illumina NovaSeq 6000 (RNA-Seq) instruments. Hi-C data were also generated from head and thorax tissue of idMerEque1 using the Arima2 kit and sequenced on the HiSeq X Ten instrument.

### Genome assembly, curation and evaluation

Assembly was carried out with Hifiasm ( Cheng *et al.*, 2021) and haplotypic duplication was identified and removed with purge_dups ( Guan *et al.*, 2020). The assembly was then scaffolded with Hi-C data ( Rao *et al.*, 2014) using YaHS ( Zhou *et al.*, 2023). The assembly was checked for contamination and corrected using the TreeVal pipeline ( Pointon *et al.*, 2023). Manual curation was performed using JBrowse2 ( Diesh *et al.*, 2023), HiGlass ( Kerpedjiev *et al.*, 2018) and Pretext ( Harry, 2022). The mitochondrial genome was assembled using MitoHiFi ( Uliano-Silva *et al.*, 2023), which runs MitoFinder ( Allio *et al.*, 2020) or MITOS ( Bernt *et al.*, 2013) and uses these annotations to select the final mitochondrial contig and to ensure the general quality of the sequence.

A Hi-C map for the final assembly was produced using bwa-mem2 ( Vasimuddin *et al.*, 2019) in the Cooler file format ( Abdennur & Mirny, 2020). To assess the assembly metrics, the *k*-mer completeness and QV consensus quality values were calculated in Merqury ( Rhie *et al.*, 2020). This work was done using Nextflow ( Di Tommaso *et al.*, 2017) DSL2 pipelines “sanger-tol/readmapping” ( Surana *et al.*, 2023a) and “sanger-tol/genomenote” ( Surana *et al.*, 2023b). The genome was analysed within the BlobToolKit environment ( Challis *et al.*, 2020) and BUSCO scores ( Manni *et al.*, 2021; Simão *et al.*, 2015) were calculated.

Table 3 contains a list of relevant software tool versions and sources.

**Table 3.** Software tools: versions and sources.

Software tool	Version	Source
BlobToolKit	4.2.1	https://github.com/blobtoolkit/blobtoolkit
BUSCO	5.3.2	https://gitlab.com/ezlab/busco
Hifiasm	0.16.1-r375	https://github.com/chhylp123/hifiasm
HiGlass	1.11.6	https://github.com/higlass/higlass
Merqury	MerquryFK	https://github.com/thegenemyers/MERQURY.FK
MitoHiFi	3	https://github.com/marcelauliano/MitoHiFi
PretextView	0.2	https://github.com/wtsi-hpag/PretextView
purge_dups	1.2.5	https://github.com/dfguan/purge_dups
sanger-tol/genomenote	v1.0	https://github.com/sanger-tol/genomenote
sanger-tol/readmapping	1.1.0	https://github.com/sanger-tol/readmapping/tree/1.1.0
TreeVal	1.0.0	https://github.com/sanger-tol/treeval
YaHS	1.2a.2	https://github.com/c-zhou/yahs

### Wellcome Sanger Institute – Legal and Governance

The materials that have contributed to this genome note have been supplied by a Darwin Tree of Life Partner. The submission of materials by a Darwin Tree of Life Partner is subject to the **‘Darwin Tree of Life Project Sampling Code of Practice’**, which can be found in full on the Darwin Tree of Life website here. By agreeing with and signing up to the Sampling Code of Practice, the Darwin Tree of Life Partner agrees they will meet the legal and ethical requirements and standards set out within this document in respect of all samples acquired for, and supplied to, the Darwin Tree of Life Project. 

Further, the Wellcome Sanger Institute employs a process whereby due diligence is carried out proportionate to the nature of the materials themselves, and the circumstances under which they have been/are to be collected and provided for use. The purpose of this is to address and mitigate any potential legal and/or ethical implications of receipt and use of the materials as part of the research project, and to ensure that in doing so we align with best practice wherever possible. The overarching areas of consideration are:

• Ethical review of provenance and sourcing of the material

• Legality of collection, transfer and use (national and international) 

Each transfer of samples is further undertaken according to a Research Collaboration Agreement or Material Transfer Agreement entered into by the Darwin Tree of Life Partner, Genome Research Limited (operating as the Wellcome Sanger Institute), and in some circumstances other Darwin Tree of Life collaborators.

## Data Availability

European Nucleotide Archive: *Merodon equestris* (narcissus bulb fly). Accession number PRJEB61688; https://identifiers.org/ena.embl/PRJEB61688 ( Wellcome Sanger Institute, 2023). The genome sequence is released openly for reuse. The *Merodon equestris* genome sequencing initiative is part of the Darwin Tree of Life (DToL) project. All raw sequence data and the assembly have been deposited in INSDC databases. The genome will be annotated using available RNA-Seq data and presented through the Ensembl pipeline at the European Bioinformatics Institute. Raw data and assembly accession identifiers are reported in Table 1.

## Author information

Members of the Natural History Museum Genome Acquisition Lab are listed here: https://doi.org/10.5281/zenodo.7139035.

Members of the University of Oxford and Wytham Woods Genome Acquisition Lab are listed here: https://doi.org/10.5281/zenodo.7125292.

Members of the Darwin Tree of Life Barcoding collective are listed here: https://doi.org/10.5281/zenodo.4893703.

Members of the Wellcome Sanger Institute Tree of Life Management, Samples and Laboratory team are listed here: https://doi.org/10.5281/zenodo.10066175.

Members of Wellcome Sanger Institute Scientific Operations: Sequencing Operations are listed here: https://doi.org/10.5281/zenodo.10043364.

Members of the Wellcome Sanger Institute Tree of Life Core Informatics team are listed here: https://doi.org/10.5281/zenodo.10066637.

Members of the Tree of Life Core Informatics collective are listed here: https://doi.org/10.5281/zenodo.5013541.

Members of the Darwin Tree of Life Consortium are listed here: https://doi.org/10.5281/zenodo.4783558.
