# Supplementary material for: The genome sequence of a hoverfly, Merodon equestris (Fabricius, 1794)
Source: Wellcome Open Res. 2024 Feb 19;9:67. [Version 1] doi: 10.12688/wellcomeopenres.20654.1 (PMC11192019; doi:10.12688/wellcomeopenres.20654.1)
Supplement: Peer review history

# Reviewer report: Reviewer response for version 1

**DOI:** 10.21956/wellcomeopenres.22859.r86064

**Published:** 2024-06-18

**Authors:** Arka Mukherjee

**Affiliations:** 1 Zoological Survey of India, Kolkata, West Bengal, India

The manuscript describes the complete genome of the bumble-mimicking hoverfly *Merodon equestris. *In an ecological context, authors may include the lacunae about the taxonomic diversity of the genus.

 The materials and methods are sufficient and a detailed explanation is mentioned in the manuscript. The assembly and annotation of the genome are perfectly presented in the graphical representation. As this work is part of the Darwin Tree of Life project, this work is adequate to assist the global biodiversity genomics.

Are sufficient details of methods and materials provided to allow replication by others?

Yes

Is the rationale for creating the dataset(s) clearly described?

Yes

Are the datasets clearly presented in a useable and accessible format?

Yes

Are the protocols appropriate and is the work technically sound?

Yes

Reviewer Expertise:

Taxonomy, DNA barcoding, Mitochondrial genome, Phylogeny, Diptera

I confirm that I have read this submission and believe that I have an appropriate level of expertise to confirm that it is of an acceptable scientific standard.

# Reviewer report: Reviewer response for version 1

**DOI:** 10.21956/wellcomeopenres.22859.r83080

**Published:** 2024-05-21

**Authors:** Antonio Ricarte

**Affiliations:** 1 University of Alicante, Alicante, Spain

The article reports about the full genome of a hoverfly species, *Merodon equestris*, of economic importance, since it causes pests in ornamental plants. In addition, the genus this species belongs to has a high species diversity in the Palaearctic Region, being one of the largest within the family in this biogeographical context.

 The article is adequately introduced and the methods and results are explained in detail. In order to give a frame to the given detailed explanation on the species *M. equestris*, authors could start the Introduction with a short paragraph on the genus *Merodon*, addressing its taxonomic diversity and poorly known larval stages and biology (e.g. Ricarte et al, 2017). In this paragraph, they may also address to the other species of the *M. equestris* group found in Europe (Vujic et al, 2021).

 Otherwise, the article is adequately written and complete in the information presented.

Are sufficient details of methods and materials provided to allow replication by others?

Yes

Is the rationale for creating the dataset(s) clearly described?

Yes

Are the datasets clearly presented in a useable and accessible format?

Yes

Are the protocols appropriate and is the work technically sound?

Yes

Reviewer Expertise:

Taxonomy and systematics; Biology; Conservation

I confirm that I have read this submission and believe that I have an appropriate level of expertise to confirm that it is of an acceptable scientific standard.

## References

[1] : A review of the early stages and host plants of the genera Eumerus and Merodon (Diptera: Syrphidae), with new data on four species. *PLoS One* .2017;12(12) : 10.1371/journal.pone.0189852 e0189852 10.1371/journal.pone.0189852 29261787 PMC5736194

[2] : Taxonomic complexity in the genus Merodon Meigen, 1803 (Diptera, Syrphidae). *Zookeys* .2021;1031: 10.3897/zookeys.1031.62125 85-124 10.3897/zookeys.1031.62125 33958908 PMC8060246
